# An Anti-Mainlobe Suppression Jamming Method Based on Improved Blind Source Separation Using Variational Mode Decomposition and Wavelet Packet Decomposition

**DOI:** 10.3390/s25113404

**Published:** 2025-05-28

**Authors:** Ruike Li, Huafeng He, Xiang Liu, Liyuan Wang, Yongquan You, Zhen Li, Xiaofei Han

**Affiliations:** College of Missile Engineering, Rocket Force Engineering University, Xi’an 710025, China

**Keywords:** variational mode decomposition, wavelet packet decomposition, blind source separation, mainlobe suppression jamming

## Abstract

Mainlobe suppression jamming significantly degrades radar detection performance. The conventional blind source separation (BSS) algorithms often fail under high-jamming-to-signal-ratio (JSR) and low-signal-to-noise-ratio (SNR) conditions. To overcome this limitation, we propose an enhanced BSS method combining variational mode decomposition (VMD) and wavelet packet decomposition (WPD), termed VMD-WPD-JADE. The proposed approach first applies VMD-WPD for noise reduction in radar signals and then utilizes the JADE algorithm to compute the separation matrix of the denoised signals, effectively achieving blind source separation of radar echoes for interference suppression. We evaluate the method using noise-amplitude modulation and noise-frequency modulation jamming scenarios. The experimental results show that at a JSR = 50 dB and an SNR = −5 dB, our method successfully separates the target signals. Compared with the conventional blind source separation (BSS) algorithms, the proposed technique demonstrates superior robustness, achieving a 4–11% improvement in the target detection probability under noise-amplitude modulation (NAM) jamming and a 4–16% enhancement under noise-frequency modulation (NFM) jamming within a signal-to-noise ratio (SNR) range of −5 dB to 5 dB.

## 1. Introduction

With the rapid development of electronic technology, the electromagnetic environment in which radar operates has become increasingly complex. The Electronic Counter-Countermeasures (ECCM) capability has emerged as a critical metric for evaluating the survivability of modern radar systems in complex electromagnetic environments [1]. Among various threats, mainlobe jamming [2,3,4,5,6], particularly mainlobe suppression jamming, has become an essential consideration in the development of radar systems. Although researchers have made progress in addressing mainlobe jamming [7,8,9], for instance, in radar waveform design, waveform diversity techniques [10] leverage multi-dimensional degrees of freedom (DoFs) in radar transmitted signals, including the time domain, the frequency domain, modulation schemes, and coding structures, to dynamically adapt to complex environments and the mission requirements, thereby enhancing systems’ capabilities in anti-jamming, target recognition, and spectral efficiency. These techniques are primarily applied in MIMO radar and synthetic aperture radar (SAR) systems. At the signal processing level, methods such as polarization filtering, blind source separation (BSS), and sparse recovery are commonly employed. However, employing any single method alone proves insufficient for effective mainlobe interference suppression. Consequently, researchers have adopted hybrid approaches combining multiple techniques for comprehensive jamming mitigation. As evidenced in reference [11], a joint transceiver design incorporating Blind Source Extraction (BSE) algorithms successfully suppressed mainlobe deceptive multi-target jamming.

Since its introduction in the 1980s, blind source separation (BSS) technology has evolved significantly, yielding numerous classical algorithms, including Fast Fixed-Point Independent Component Analysis (FastICA) [12,13,14,15,16], Second-Order Blind Identification (SOBI) [17,18,19], and joint approximate diagonalization of eigenmatrices (JADE). These algorithms primarily differ in their cost functions and optimization approaches. Among them, the JADE algorithm has been widely adopted and further improved by researchers due to its superior separation performance and low computational complexity. This method does not require prior knowledge of the signals or the mixing process, yet it can separate the original independent source signals from multiple observed signals. Reference [20] developed a multi-domain (spatial, temporal, and polarization) anti-jamming approach combining BSS to counter composite mainlobe jamming. Reference [21] introduced a hybrid polarization radar anti-SMSP jamming algorithm based on jamming reconstruction and BSS. Compared with traditional MSNR-BSS algorithms, this approach reduced the computational complexity while maintaining effective jamming suppression even at a jamming-to-signal ratio (JSR) of 25 dB. Reference [22] proposed an improved BSS algorithm based on a Frequency-Domain Robust Principal Component Analysis (FD-RPCA-BSS), which effectively suppressed mainlobe jamming in rotating array radars. Reference [23] employed the JADE algorithm in a frequency diverse array (FDA)-MIMO radar system to separate the target signals from mainlobe repeater jamming.

Nevertheless, the traditional research has not adequately accounted for noise. In practical scenarios, a radar’s ability to detect targets is often compromised by environmental noise. A critical limitation of the JADE algorithm, for instance, is its deteriorating performance under low-signal-to-noise-ratio (SNR) conditions. Reference [24] proposed an improved BSS-based anti-jamming algorithm for spread spectrum communications, which maintains a robust separation performance at low SNRs. However, it requires iterative calculations to accumulate the separation matrix, and the number of accumulations significantly impacts the algorithmic performance. Reference [25] proposed a hybrid method combining BSS with a singular spectrum analysis (SSA) for radar signal recognition, achieving successful separation at a 5 dB SNR after SSA-based denoising but without exploring more challenging sub-0 dB conditions. Reference [26] improved the MSNR-BSS algorithm by incorporating wavelet threshold denoising, which successfully separated diffuse jamming in frequency diverse array multiple-input multiple-output (FDA-MIMO) radar systems under high-SNR conditions. But this method demonstrated a low resolution in high-frequency bands, potentially leading to the partial loss of information on the target.

To address the performance degradation of traditional blind source separation (BSS) algorithms under high-jamming-to-signal-ratio (JSR) and low-signal-to-noise-ratio (SNR) conditions in radar systems subjected to mainlobe suppression jamming, this paper proposes an improved BSS method based on variational mode decomposition and wavelet packet decomposition (VMD-WPD) for anti-mainlobe suppression jamming. The main innovations of this work are as follows:

(1) A novel hybrid denoising methodology combining variational mode decomposition (VMD) and wavelet packet decomposition (WPD). The proposed VMD-WPD method first utilizes VMD’s adaptive frequency band segmentation capability to overcome the end-effect limitations of conventional EMD techniques. Subsequently, it applies WPD-based adaptive thresholding to the decomposed modal functions, enabling effective noise reductions across all frequency bands. This integrated approach simultaneously suppresses both the global and local noise components in radar echo signals while preserving critical target information.

(2) An enhanced threshold model is developed to mitigate the pseudo-Gibbs oscillations induced by hard thresholding discontinuities in wavelet packet denoising. The proposed solution integrates a sigmoid function to smoothly approximate the threshold transition, effectively suppressing pseudo-oscillations in the reconstructed signals while maintaining essential components of the signal. This approach significantly improves the signal fidelity compared to that with the conventional thresholding methods.

(3) A separation matrix correction method based on denoising optimization is proposed. By constructing an optimized fourth-order cumulant matrix model for the denoised signals, the method effectively compensates for matrix distortion caused by noise. Compared to the conventional JADE-BSS method, the proposed approach demonstrates 4% and 8% improvements in successful target signal separation under noise-amplitude modulation (NAM) and noise-frequency modulation (NFM) suppression jamming, respectively.

The remainder of this paper is organized as follows: Section 2 presents the array signal reception model. Section 3 provides a detailed derivation of the proposed mainlobe interference suppression approach, including both the JADE-BSS method and the VMD-WPD-JADE method. Section 4 presents comprehensive simulations to validate the proposed method, along with an analysis of the computational complexity of each module, followed by the conclusions in Section 5.

## 2. The Array Signal Reception Model

Consider a radar mainlobe jamming scenario comprising one T/R radar, one jammer, and one target. The radar is a uniform linear array with *M* elements, located at $\left(x_{R},y_{R},z_{R}\right)$. Both the jammer and the target are in the far-field region, with the stationary target assumed to be at $\left(x_{T},y_{T},z_{T}\right)$ and the stationary jammer at $\left(x_{J},y_{J},z_{J}\right)$. The radar transmits the signal $s_{T}\left(t\right)$, while the jammer emits the high-power barrage jamming signal $s_{J}\left(t\right)$, with Doppler information not considered. The target echo signal $s_{J}\left(t\right)$ received by the *m*-th element of the radar array is

(1) $$s_{m}^{RT}\left(t\right)=s_{T}\left(t-\tau_{T}\right)\exp\left(-j2\pi \frac{(m-1)d}{\lambda }\sin\theta_{T}\right),m=1,2,\ldots ,M$$

where $\tau_{T}=2\sqrt{{\left(x_{R}-x_{T}\right)}^{2}+{\left(y_{R}-y_{T}\right)}^{2}+{\left(z_{R}-z_{T}\right)}^{2}}/c$ is the propagation time delay of the target signal. $c=3\times 10^{8}$ m/s represents the propagation speed of electromagnetic waves. *d* is the inter-element spacing. λ is the wavelength of the electromagnetic wave, and $\theta_{T}$ is the direction of arrival of the target signal.

Similarly, the jamming signal $s_{m}^{RJ}\left(t\right)$ received by the m-th radar array element is

(2) $$s_{m}^{RJ}\left(t\right)=s_{T}\left(t-\tau_{J}\right)\exp\left(-j2\pi \frac{(m-1)d}{\lambda }\sin\theta_{J}\right),m=1,2,\ldots ,M$$

where $\tau_{J}=2\sqrt{{\left(x_{R}-x_{J}\right)}^{2}+{\left(y_{R}-y_{J}\right)}^{2}+{\left(z_{R}-z_{J}\right)}^{2}}/c$ is the propagation time delay of the jamming signal, and $\theta_{J}$ is the direction of arrival of the jamming signal. Therefore, the echo signal $r_{m}\left(t\right)$ received by the *m*-th radar array element can be expressed as

(3) $$r_{m}\left(t\right)=s_{m}^{RT}\left(t\right)+s_{m}^{RJ}\left(t\right)+n_{m}\left(t\right)$$

where $n_{m}\left(t\right)$ represents additive white Gaussian noise, m = 1, 2, …, *M*.

## 3. An Improved Mainlobe Suppression Jamming Resistance Method Based on VMD-WPD Enhanced Blind Source Separation

### 3.1. JADE-BSS

The JADE-BSS algorithm offers significant advantages for radar mainlobe interference suppression, including its blind operation (requiring no prior information) and high computational efficiency [27]. This algorithm obtains an effective estimation of the mixing matrix in the blind source separation system by jointly diagonalizing a set of feature matrices. The implementation process mainly includes signal preprocessing, calculation of the fourth-order cumulant matrix, joint diagonalization of the feature matrices, and signal separation [28].

Step 1: Signal preprocessing

The radar echo signal r(t) is first centered by subtracting its mean value to obtain zero-mean normalization, yielding the processed signal $r^{\prime }\left(t\right)$. The zero-mean signal $r^{\prime }\left(t\right)$ then undergoes whitening processing to remove correlations. This involves the following: (1) Computing the covariance matrix $C_{x}=E\left\{r^{\prime }\left(t\right)r^{\prime H}\left(t\right)\right\}$. (2) Performing eigenvalue decomposition $C_{x}=U\Lambda U^{H}$ to obtain the eigenvector matrix *U* and the diagonal eigenvalue matrix Λ. (3) Calculating the whitening matrix $W=\Lambda^{-1/2}U^{H}$. (4) Generating the whitened signal $z\left(t\right)=Wr^{\prime }\left(t\right)$.

Step 2: Constructing the fourth-order cumulant matrix

Define the fourth-order cumulant matrix $Q_{z}\left(T\right)$ of the whitened signal z(t) [29], where its (i,j)-th element is given by

(4) $${\left[Q_{z}\left(T\right)\right]}_{ij}=\sum_{k=1}^{N}\sum_{l=1}^{N}\mathrm{cum}\left(z_{i}\left(t\right),z_{j}^{*}\left(t\right),z_{k}\left(t\right),z_{l}^{*}\left(t\right)\right)m_{kl}$$

where $\mathrm{cum}(z_{i}\left(t\right),z_{j}^{*}\left(t\right),z_{k}\left(t\right),z_{l}^{*}\left(t\right))$ denotes the fourth-order cumulant of the signal z(t). $m_{kl}$ represents the (*k*,*l*)-th element of an arbitrary N×N dimensional matrix ***T***. The asterisk (*) denotes complex conjugation.

Step 3: Joint diagonalization of characteristic matrices

Applying eigenvalue decomposition to the fourth-order cumulant matrix yields the unitary matrix ***V*** [30], expressed as

(5) $$Q_{z}\left(T\right)=V\Sigma V^{H}$$

where Σ is the diagonal matrix of the fourth-order cumulant matrix.

Finally, the source signals after blind source separation can be obtained from the matrix ***V***, as shown in Equation (6).

(6) $$s\left(t\right)=V^{H}Wr^{\prime }\left(t\right)$$

**Figure 1:**
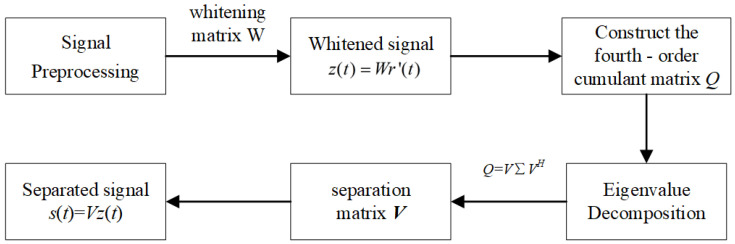
Flowchart of JADE-BSS algorithm’s implementation.

The principle of the JADE-BSS algorithm is illustrated in Figure 1 below:

As is evident from the whitening process in the JADE-BSS algorithm, the whitening matrix ***W*** depends on the second-order statistics of the signal $r^{\prime }\left(t\right)$:

(7) $$C_{x}=E\left\{r^{\prime }\left(t\right)r^{H}\left(t\right)\right\}=AC_{s}A^{H}+\sigma^{2}I$$

where A is the mixing matrix, $C_{s}$ is the covariance matrix of the source signals, and σ represents the noise power.

Under conditions of a high jam-to-signal ratio (JSR) and a low signal-to-noise ratio (SNR), when $\sigma^{2}\gg ∥AC_{s}A^{H}∥$, noise dominates the covariance matrix. This results in the whitened signal z(t) failing to effectively preserve the target signal information. Consequently, the fourth-order cumulants of z(t) become contaminated by noise, causing the jointly diagonalized matrix Σ to lose its ’diagonalizability’. This ultimately leads to convergence to an incorrect separation matrix ***V*** during the optimization process, resulting in separation failure.

To address this, this paper incorporates the VMD-WPD algorithm into the JADE framework. The approach first applies noise suppression to the radar echo signals to reduce the interference of noise in the whitened signals and optimize the separation matrix. The enhanced separation matrix then effectively separates the target signals from the interference signals.

### 3.2. VMD-WPD-JADE

VMD-WPD is a signal processing method that combines variational mode decomposition [31,32,33,34,35,36] with wavelet packet threshold denoising [37,38]. The core of VMD lies in establishing dual constraints: minimizing the total estimated bandwidth of all modal components, while ensuring exact reconstruction of the original input signal through modal superposition [39]. This algorithm demonstrates significant advantages when processing non-stationary time series with high complexity and strong nonlinearity. Assuming the signal rt is decomposed into *K* modal components, the variational model is constructed as shown in Equation (8).

(8) $$\begin{array}{cc} \; & \min_{\left(u_{k},\omega_{k}\right)}\left\{\sum_{k}{∥\partial_{t}\left[\left(\delta \left(t\right)+\frac{j}{\pi t}\right)*u_{k}\left(t\right)\right]e^{-j\omega_{k}t}∥}_{2}^{2}\right\}\\ \; & s.t.\;\sum_{k}u_{k}=r\left(t\right) \end{array}$$

where *t* represents time, and $\omega_{k}$ denotes the actual center frequency of the *k*-th modal component. $e^{-j\omega_{k}t}$ indicates the estimated center frequency. δ(t) is the Dirac delta function. $u_{k}\left(t\right)$ corresponds to the *k*-th mode function. ${∥\cdot ∥}_{2}^{2}$ signifies the L2 norm.

By leveraging the advantages of quadratic penalty terms and the Lagrange multiplier method, the introduction of an augmented Lagrangian function transforms the aforementioned constrained variational problem into an unconstrained one, as shown in Equation (9):

(9) $$\begin{array}{cc} L(\left\{u_{k}\right\},\left\{\omega_{k}\right\},\lambda ) & =\alpha \sum_{k}{∥\partial_{t}\left[\left(\delta \left(t\right)+\frac{j}{\pi t}\right)*u_{k}\left(t\right)\right]e^{-j\omega_{k}t}∥}_{2}^{2}\\ \; & +\;{∥s\left(t\right)-\sum_{k}u_{k}\left(t\right)∥}_{2}^{2}+\langle \lambda \left(t\right),s\left(t\right)-\sum_{k}u_{k}\left(t\right)\rangle \end{array}$$

where λ is the Lagrangian multiplier, α denotes the penalty parameter, and 〈·,·〉 represents the inner product operator.

Optimization is performed using the method of the multiplier iterative algorithm, with the updates for the mode functions and the center frequencies given by Equations (10) and (11), respectively:

(10) $${\hat{u}}_{k}^{n+1}\left(\omega \right)=\frac{s\left(\omega \right)-\sum_{i=1,i<k}^{K}{\hat{u}}_{i}^{n+1}\left(\omega \right)-\sum_{i=1,i>k}^{K}{\hat{u}}_{i}^{n}\left(\omega \right)+\frac{{\hat{\lambda }}^{n}\left(\omega \right)}{2}}{1+2\alpha {\left(\omega -\omega_{k}^{n}\right)}^{2}}$$

(11) $$\omega_{k}^{n+1}=\frac{\int_{0}^{\infty }\omega {\left|{\hat{u}}_{k}^{n+1}\left(\omega \right)\right|}^{2}d\omega }{\int_{0}^{\infty }{\left|{\hat{u}}_{k}^{n+1}\left(\omega \right)\right|}^{2}d\omega }$$

where *n* is the iteration index. ${\hat{u}}_{k}^{n+1}\left(\omega \right)$, s(ω), and ${\hat{\lambda }}^{n}\left(\omega \right)$ denote the Fourier transforms of ${\hat{u}}_{k}^{n+1}\left(t\right)$, s(t), and ${\hat{\lambda }}^{n}\left(t\right)$, respectively.

The iterative process continues by successively updating the functional until the convergence criterion in Equation (12) is satisfied.

(12) $$\sum_{k=1}^{K}\frac{∥{\hat{u}}_{k}^{n+1}-{\hat{u}}_{k}^{n}{∥}_{2}^{2}}{∥{\hat{u}}_{k}^{n}{∥}_{2}^{2}}<\gamma$$

where γ is the convergence tolerance.

Through iterative optimization, variational mode decomposition is applied to the signal received by each array element. Assume the received signal $r_{m}\left(t\right)$ of the *m*-th array element is decomposed into *K* modal components, as shown in Equation (13):

(13) $$r_{m}\left(t\right)=\sum_{k=1}^{K}u_{k}^{m}\left(t\right)$$

where $u_{k}^{m}\left(t\right)$ represents the Intrinsic Mode Function (IMF) component, k=1,2,…,K, m=1,2,…,M, *M* denotes the number of array elements.

Wavelet packet decomposition (WPD) is an advanced signal processing technique that extends a conventional wavelet analysis. This method offers superior time-frequency localization capabilities, enabling simultaneous characterization of both low-frequency and high-frequency signal components across the entire bandwidth [40]. The WPD algorithm effectively reduces noise through three key steps: initial multi-scale signal decomposition, followed by adaptive thresholding of the wavelet coefficients, and concluding with signal reconstruction through inverse transformation. Compared to the standard wavelet methods, WPD provides more precise frequency band partitioning while maintaining essential features of the signal, resulting in significantly improved signal-to-noise ratios. The complete decomposition–reconstruction process ensures optimal preservation of critical characteristics of the signal during noise suppression. The modal component signal is decomposed into a full sub-band tree structure, as shown in Equation (14):

(14) $$u_{k}^{m}\left(t\right)=\sum_{j=1}^{J}\sum_{i=0}^{2^{j}-1}d_{j,i}^{m}\psi_{j,i}^{m}\left(t\right)$$

where *J* represents the decomposition level. *i* denotes the sub-band index. $\psi_{j,i}\left(t\right)$ corresponds to the wavelet packet basis function. $d_{j,i}$ indicates the wavelet packet coefficient.

The wavelet packet coefficients undergo thresholding processing. Conventional thresholding includes hard and soft methods: hard thresholding causes signal oscillations due to discontinuity at the threshold point, while soft thresholding introduces a fixed bias into the coefficients [41]. To address these limitations, a sigmoid function is introduced as a smooth transition [42], constructing a novel thresholding function:

(15) $${\hat{d}}_{j,i}^{m}\left(t\right)=d_{j,i}^{m}\left(t\right)\cdot S\left(d_{j,i}^{m}\left(t\right)\right)=\frac{d_{j,i}^{m}\left(t\right)}{1+e^{-\alpha (|d_{j,i}^{m}\left(t\right){|}^{\lambda }-1)}}$$

where ${\hat{d}}_{j,i}^{m}\left(t\right)$ represents the processed wavelet packet coefficient. $S\left(\cdot \right)=\frac{1}{1+e^{-\alpha (|\cdot {|}^{\lambda }-1)}}$ denotes the sigmoid function. α is the smoothing factor. The threshold $\lambda =\sigma \sqrt{2\log N}$, where σ is the standard deviation in the signal ($\sigma =\mathrm{Median}(|d_{j,i}^{m}\left(t\right)|)/0.6745$).

The thresholding function is defined piecewise as follows:

(16) $${\hat{d}}_{j,i}^{m}\left(t\right)=\left\{\begin{array}{cc} d_{j,i}^{m}\left(t\right), & |d_{j,i}^{m}\left(t\right)|\gg \lambda \\ d_{j,i}^{m}\left(t\right)\cdot \left(\frac{1}{2}+\frac{\alpha }{4\lambda }\left(\right|d_{j,i}^{m}\left(t\right)\left|-\lambda \right)\right), & |d_{j,i}^{m}\left(t\right)|\approx \lambda \\ 0, & |d_{j,i}^{m}\left(t\right)|\ll \lambda \end{array}\right.$$

**Figure 2:**
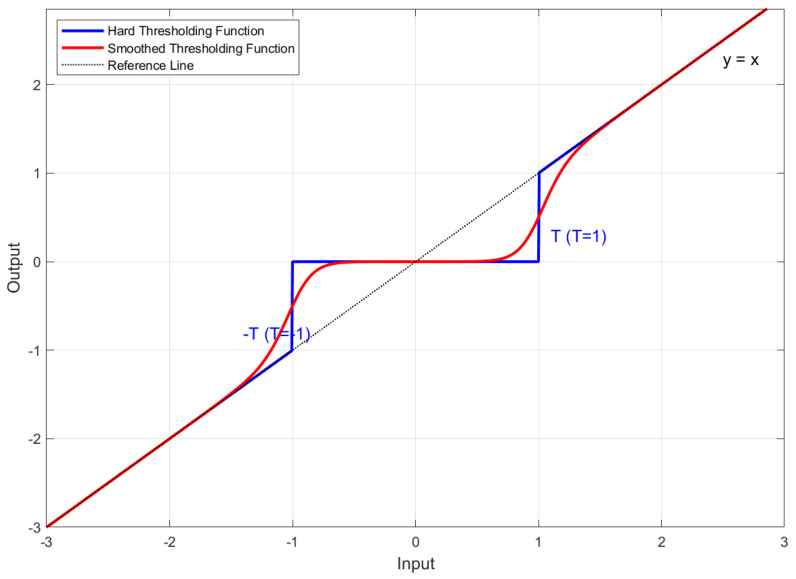
Comparison of thresholding functions before and after improvement.

With the smoothing factor set to α=15, Figure 2 compares the proposed and conventional thresholding functions. Unlike traditional threshold functions that exhibit discontinuity at the threshold point ±T, the improved function achieves a continuous transition near ±T, effectively suppressing oscillations in the denoised signals.

Using the thresholded coefficients ${\hat{d}}_{j,i}^{m}\left(t\right)$, the signal is reconstructed to obtain the denoised modal component ${\hat{u}}_{k}^{m}\left(t\right)$:

(17) $${\hat{u}}_{k}^{m}\left(t\right)=\sum_{j=1}^{J}\sum_{i=0}^{2^{j}-1}{\hat{d}}_{j,i}^{m}\psi_{j,i}^{m}\left(t\right)$$

Assuming additive Gaussian noise n(t) with the power $\sigma^{2}$, the noise power in the radar echo signal is reduced after denoising via the VMD-WPD algorithm:

(18) $$\sigma_{VMD-WPD}^{2}=\beta \sigma_{n}^{2},\beta <1$$

where $\sigma_{VMD-WPD}^{2}$ represents the noise power after denoising. β is the noise power attenuation coefficient. The value of β depends on the decomposition level in VMD, the penalty factor, and the threshold selection strategy.

The signal-to-noise ratio (SNR) after VMD-WPD processing is given by

(19) $$SNR_{VMD-WPD}=SNR+10\log_{10}\left(\frac{1}{\beta }\right)$$

As is evident from the above formulation, the VMD-WPD algorithm significantly enhances the signal-to-noise ratio. The final denoised signal $\hat{r}\left(t\right)$ is obtained by summing all processed modal components:

(20) $$\hat{r}\left(t\right)=\sum_{m=1}^{M}\sum_{k=1}^{K}{\hat{u}}_{k}^{m}\left(t\right)$$

Using the JADE algorithm, a new separation matrix ***U*** is constructed based on the denoised signal $\hat{r}\left(t\right)$. The target signal $\hat{s}\left(t\right)$ is then extracted through the following operation:

(21) $$\hat{s}\left(t\right)=U^{H}z\left(t\right)$$

**Figure 3:**
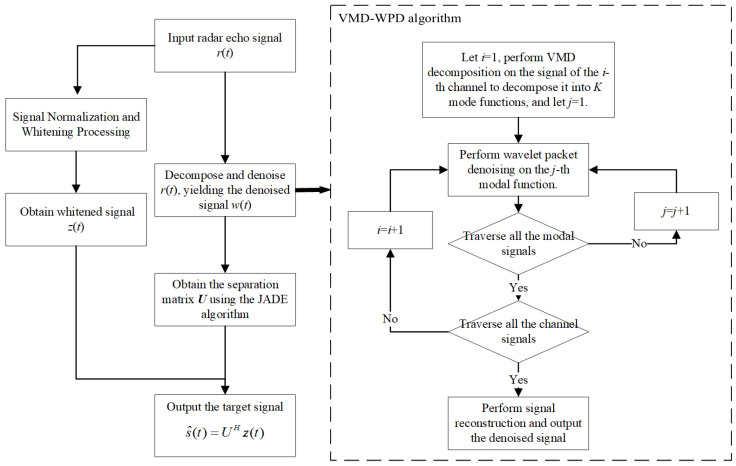
Flowchart of VMD-WPD-JADE methodology.

The flowchart for implementation of the method is shown in Figure 3.

## 4. Simulation Analysis

This study focuses on two representative types of suppression jamming: noise-amplitude modulation (NAM) jamming and noise-frequency modulation (NFM) jamming. These particular interference types were selected for investigation because they represent the most prevalent and challenging forms of mainlobe suppression jamming encountered in modern radar systems. The NAM jamming technique generates broadband interference through amplitude modulation of noise signals, while NFM jamming creates its suppressive effects using frequency-modulated noise waveforms. Both jamming types can significantly degrade the radar detection performance through distinct physical mechanisms, making them ideal test cases for evaluating the robustness of anti-jamming algorithms.

**Figure 4:**
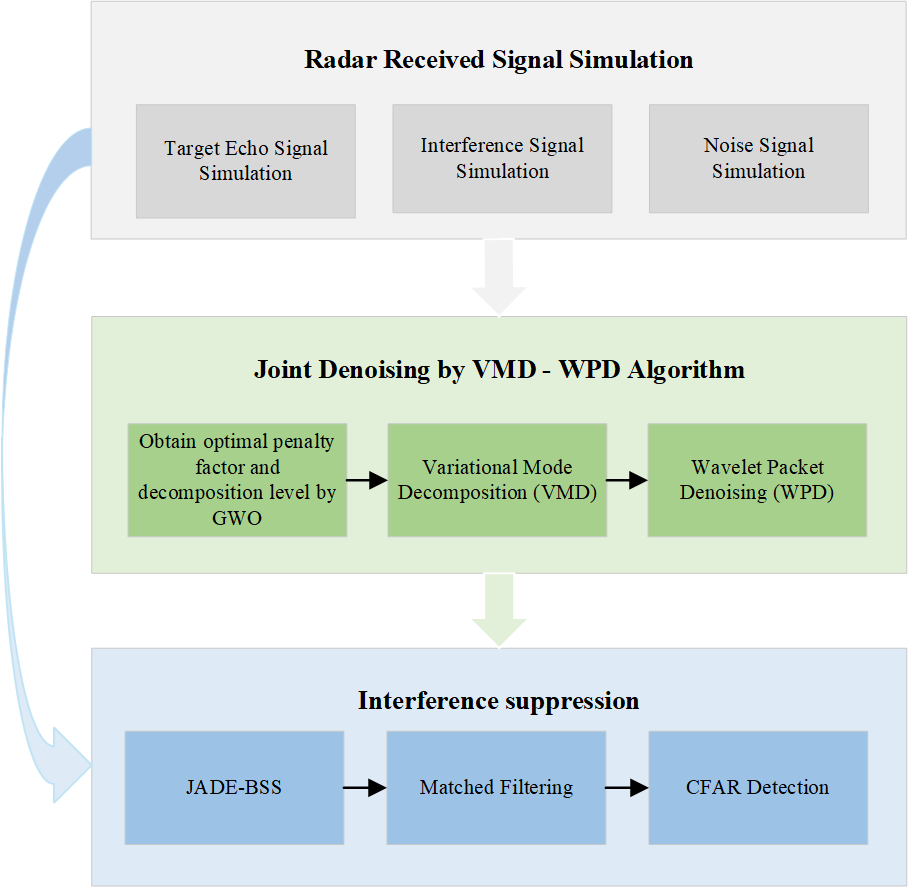
Flowchart of anti-suppression jamming operation.

Using MATLAB R2023b software, we simulate the radar received signals under varying SNR conditions. The simulated signals undergo blind source separation (BSS) through both the proposed method and conventional JADE-BSS. Subsequently, matched filtering is applied to the separated signals. Monte Carlo simulations are conducted to calculate and compare the detection probability and the peak-to-sidelobe ratio (PSLR) [43] for both methods, evaluating their anti-jamming performance. The operational flowchart is presented in Figure 4.

### 4.1. Parameter Configuration

Configure a uniform linear array radar system with a transmit–receive capability, comprising six array elements spaced at half-wavelength intervals. The radar parameters are specified in Table 1, with the transmitted linear-frequency-modulated (LFM) signal given by Equation (22):

(22) $$s_{T}\left(t\right)=\exp\left\{j2\pi \left(f_{c}t+\frac{1}{2}kt^{2}\right)\right\},t\in \left(0,T_{p}\right]$$

Assume the radar receives one target signal and one amplitude-modulated noise jamming signal, with the jammer located near the target. The directions of arrival (DOAs) are 1° and 2°, respectively. The spatial coordinates of the radar, the target, and the jammer are listed in Table 2 (units: meters).

The amplitude-modulated noise jamming signal is expressed as

(23) $$J\left(t\right)=\left(U_{0}+U_{n}\left(t\right)\right)\exp\left(j\left(2\pi f_{j}t+\varphi \right)\right)$$

where $U_{n}\left(t\right)$ represents zero-mean Gaussian white noise with unit variance. $f_{j}$ = 16 GHz is the jamming carrier frequency. φ denotes a uniformly distributed random variable over [0,2π).

The noise-frequency modulation (NFM) jamming signal is expressed as

(24) $$J_{f}\left(t\right)=U_{j}\cos\left(2\pi f_{c}t+2\pi K_{F}\int_{0}^{t}u\left(\tau \right)d\tau +\varphi_{f}\right)$$

where $U_{j}$ is the amplitude of the jamming signal. $f_{c}$ is the carrier center frequency. $K_{F}$ is the frequency modulation slope. u(τ) represents the modulating noise, which is a generalized stationary random process with zero mean. $\varphi_{f}$ is a random variable uniformly distributed over [0,2π) and is statistically independent of u(τ).

**Figure 5:**
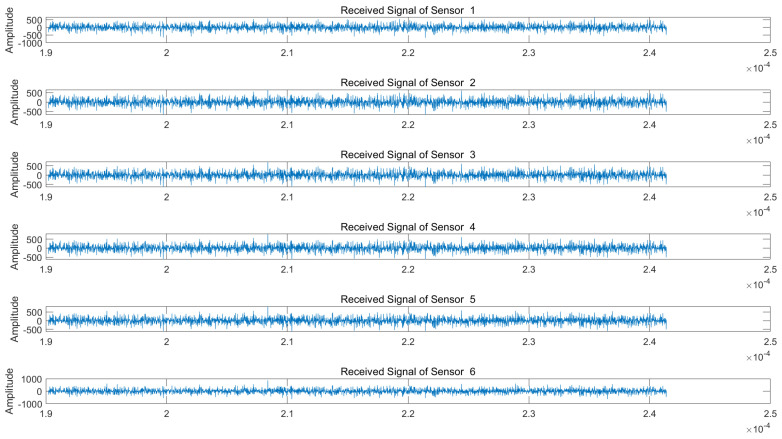
Echo signals received by radar array elements under NAM suppression jamming.

**Figure 6:**
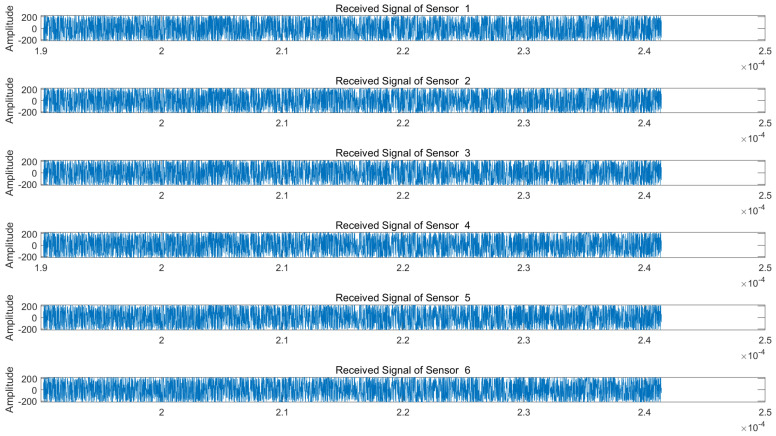
Echo signals received by radar array elements under NFM suppression jamming.

We assume a jam-to-signal ratio (JSR) of 50 dB and a signal-to-noise ratio (SNR) of 5 dB. Under both NAM and NFM suppression jamming scenarios, the signals received by each array element of the radar are illustrated in Figure 5 and Figure 6, respectively.

As is evident from Figure 5 and Figure 6, the target echo signal is completely submerged within the barrage jamming signal, rendering target identification impossible. We subsequently perform blind source separation (BSS) using the conventional JADE algorithm and the proposed VMD-WPD-JADE algorithm for a comparative analysis.

### 4.2. Parameter Selection for the VMD-WPD Algorithm

Regarding variational mode selection, the penalty factor α and the decomposition level *K* directly impact the signal decomposition performance. An insufficient α value leads to excessive mode bandwidths causing modal aliasing, while an excessive α value results in loss of details on the signal. Insufficient *K* values cause under-decomposition, failing to extract critical signal features, whereas excessive *K* values increase the computational complexity and may introduce noise-dominated modes, reducing the interpretability. Using a 5 dB SNR scenario as an example, we employ the grey wolf optimizer (GWO) [44,45] to optimize the parameters for each array element’s received signal. After 30 iterations, the optimal decomposition levels and penalty factors are obtained as shown in Table 3:

To validate that the penalty factor (α) and the decomposition level (*K*) obtained through GWO optimization represent the optimal parameters, this study analyzes the signals received by the first array element channel under noise-amplitude modulation suppression jamming with an SNR = 5 dB and a JNR = 50 dB. Four suboptimal parameter combinations were evaluated:

(1) An overestimated penalty factor (α = 10,000, *K* = 15);
(2) An underestimated penalty factor (α = 3000, *K* = 15);
(3) Excessive decomposition levels (α = 6492, *K* = 20);
(4) Insufficient decomposition levels (α = 6492, *K* = 10).

The denoising performance was quantified using the normalized mean square error (NMSE) between the pre- and post-processing signals, as detailed in Table 4.

From Table 4, it can be observed that the NMSE between the denoised and original signals for all four suboptimal parameter combinations is higher than that of the optimal combination (α = 6492, *K* = 15). This indicates that the signal distortion after denoising is lower when using the optimized parameters (α = 6492, *K* = 15) compared to the other four suboptimal configurations, thereby validating the reliability of the proposed optimization results.

**Figure 7:**
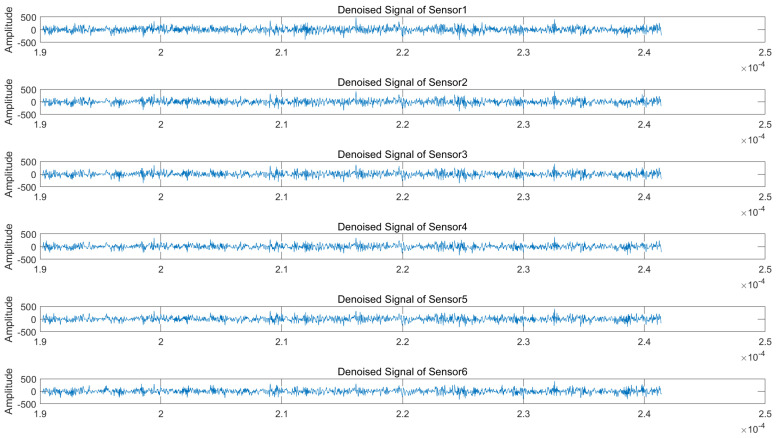
Denoised received signals at radar array elements (NAM suppression jamming scenario).

**Figure 8:**
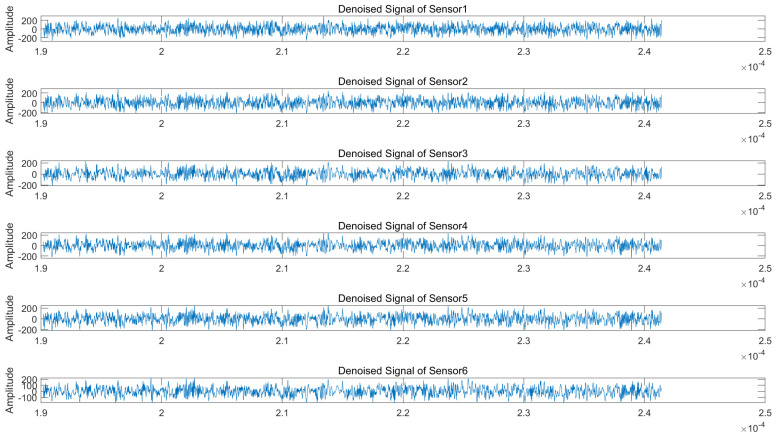
Denoised received signals at radar array elements (NFM suppression jamming scenario).

Regarding the wavelet packet denoising parameters, we select the sym9 function as the wavelet basis due to its superior symmetry properties, which are particularly suitable for processing non-stationary signals. The decomposition level is set to 3, as higher levels would significantly increase the computational complexity while potentially introducing noise artifacts. Figure 7 and Figure 8 display the time-domain waveforms of the denoised signals received by each array element under 50 dB jamming-to-signal ratio (JSR) and 5 dB signal-to-noise ratio (SNR) conditions. The results demonstrate noticeable reductions in signal glitches across all channels, confirming effective noise suppression. Subsequent processing involves applying blind source separation (BSS) to both the original and denoised signals, followed by matched filtering of the two separated source signals.

### 4.3. The Signal Separation Performance Under Multiple SNR Conditions

This study focuses on noise-frequency modulation (NFM) jamming and noise-amplitude modulation (NAM) jamming as research subjects. Under conditions with a jamming-to-signal ratio (JSR) of 50 dB and signal-to-noise ratios (SNRs) of −5 dB, 0 dB, and 5 dB, respectively, the joint approximate diagonalization of eigenmatrices (JADE) blind source separation (BSS) method and the proposed method are employed to apply BSS to radar received signals. Subsequently, pulse compression and constant false alarm rate (CFAR) detection are conducted for the BSS-processed signals. Finally, Monte Carlo simulations are performed within an SNR range of −5 dB to 5 dB, and the effectiveness of the proposed method is verified using two key metrics: the detection probability (Pd) and the peak-to-sidelobe ratio (PSLR).

(a) Noise-amplitude modulation (NAM) jamming

(1) Separation performance at an SNR = −5 dB

**Figure 9:**
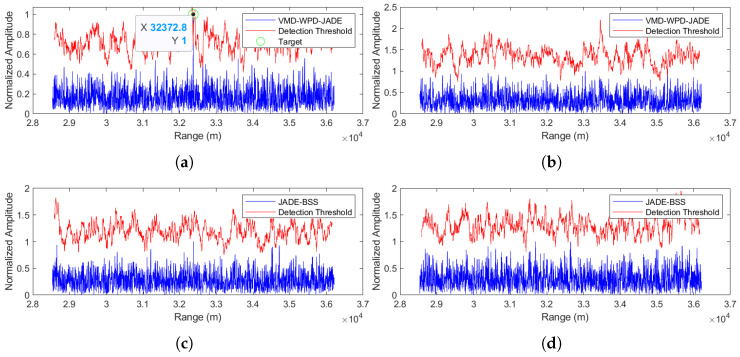
Comparison of anti-noise-amplitude modulation jamming performance between the proposed method and JADE-BSS under −5 dB signal-to-noise ratio (SNR) conditions. (**a**) Signal 1 separated by the VMD-WPD-JADE algorithm. (**b**) Signal 2 separated by the VMD-WPD-JADE algorithm. (**c**) Signal 1 separated by the JADE-BSS algorithm. (**d**) Signal 2 separated by the JADE-BSS algorithm.

**Figure 10:**
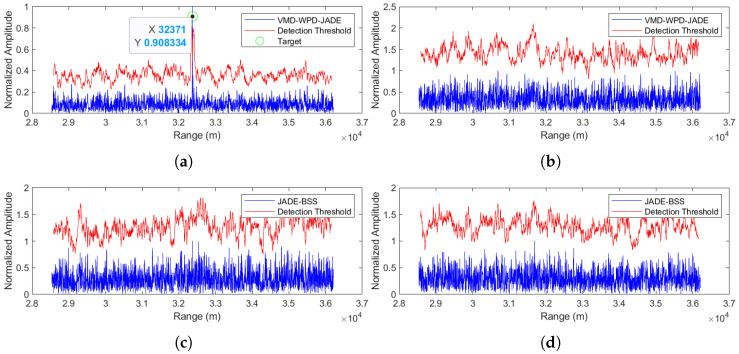
Comparison of anti-noise-amplitude modulation jamming performance between the proposed method and JADE-BSS under 0 dB signal-to-noise ratio (SNR) conditions. (**a**) Signal 1 separated by the VMD-WPD-JADE algorithm. (**b**) Signal 2 separated by the VMD-WPD-JADE algorithm. (**c**) Signal 1 separated by the JADE-BSS algorithm. (**d**) Signal 2 aeparated by the JADE-BSS algorithm.

**Figure 11:**
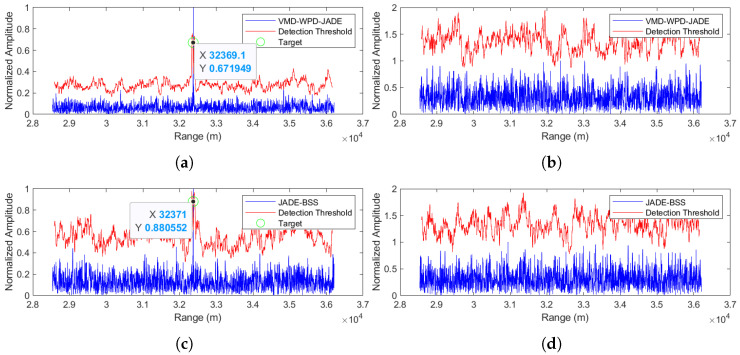
Comparison of anti-noise-amplitude modulation jamming performance between the proposed method and JADE-BSS under 5 dB signal-to-noise ratio (SNR) conditions. (**a**) Signal 1 separated by the VMD-WPD-JADE algorithm. (**b**) Signal 2 separated by the VMD-WPD-JADE algorithm. (**c**) Signal 1 separated by the JADE-BSS algorithm. (**d**) Signal 2 separated by the JADE-BSS algorithm.

**Figure 12:**
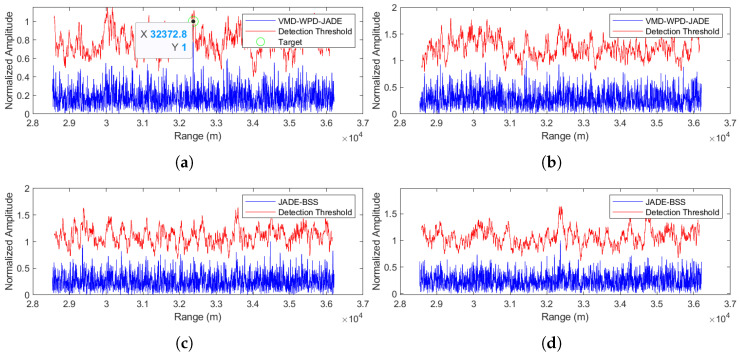
Comparison of anti-noise-frequency modulation jamming performance between the proposed method and JADE-BSS under −5 dB signal-to-noise ratio (SNR) conditions. (**a**) Signal 1 separated by the VMD-WPD-JADE algorithm. (**b**) Signal 2 separated by the VMD-WPD-JADE algorithm. (**c**) Signal 1 separated by the JADE-BSS algorithm. (**d**) Signal 2 separated by the JADE-BSS algorithm.

**Figure 13:**
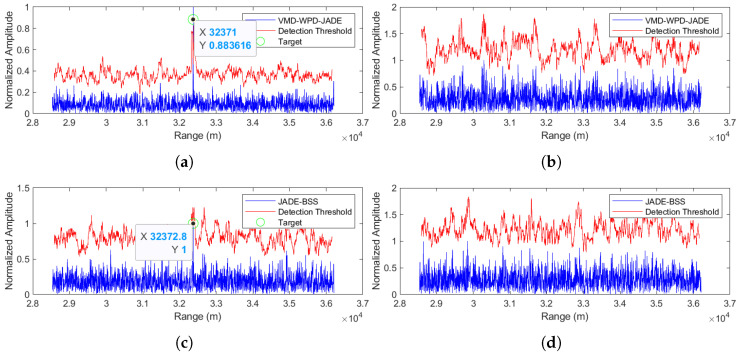
Comparison of anti-noise-frequency modulation jamming performance between the proposed method and JADE-BSS under 0 dB signal-to-noise ratio (SNR) conditions. (**a**) Signal 1 separated by the VMD-WPD-JADE algorithm. (**b**) Signal 2 separated by the VMD-WPD-JADE algorithm. (**c**) Signal 1 separated by the JADE-BSS algorithm. (**d**) Signal 2 separated by the JADE-BSS algorithm.

**Figure 14:**
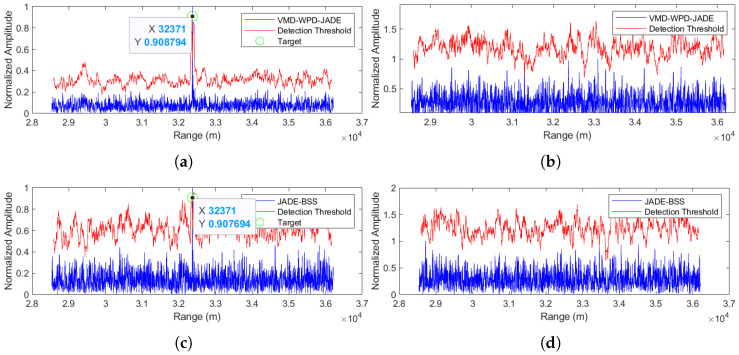
Comparison of anti-noise-frequency modulation jamming performance between the proposed method and JADE-BSS under 5 dB signal-to-noise ratio (SNR) conditions. (**a**) Signal 1 separated by the VMD-WPD-JADE algorithm. (**b**) Signal 2 separated by the VMD-WPD-JADE algorithm. (**c**) Signal 1 separated by the JADE-BSS algorithm. (**d**) Signal 2 separated by the JADE-BSS algorithm.

The separation results for the proposed method are shown in Figure 9a,b, while JADE-BSS’s results appear in Figure 9c,d. In the figures, the blue curve represents the radar echo signal after pulse compression, while the red curve indicates the threshold level of constant false alarm rate (CFAR) detection with the false alarm probability set to 0.02. A target signal is considered detected at any point where the amplitude of the blue signal exceeds the corresponding threshold value of the red curve. (Unless otherwise specified, Figure 9, Figure 10, Figure 11, Figure 12, Figure 13 and Figure 14 follow the same representation convention.) At a −5 dB SNR, JADE-BSS fails to extract the target signal, whereas our method successfully separates the target with the detected range at 32,372.8 m. Given the radar coordinates [8000, 28,000, 30,000] (in meters) and the true target position [10,000, 40,000, 0], the theoretical radial distance is calculated as L ≈ 32,372.8 m. This shows that, in this case, the method proposed in this paper has a very high positioning accuracy.

(2) Separation performance at an SNR = 0 dB

The separation results for the proposed method are shown in Figure 10a,b, while Figure 10c,d display the JADE-BSS method’s performance. The simulation results demonstrate that the proposed method maintains effective target signal separation even at a 0 dB SNR. The detected target range is 32,371 m from the radar, with a mere 1.8 m positioning error, showing excellent agreement with the ground truth.

(3) Separation performance at an SNR = 5 dB

The comparative analysis of the separation performance at a 5 dB SNR (Figure 11a,b for the proposed method versus Figure 11c,d for JADE-BSS) demonstrates that while both methods can detect the target signal, the proposed method achieves significantly lower noise levels in the separated target signal.

From Figure 9a, Figure 10a and Figure 11a, the observed localization errors when applying the proposed method appear to exhibit an increasing trend with higher SNR values (−5 dB, 0 dB, and 5 dB). To investigate this phenomenon systematically, we conducted 100 Monte Carlo simulations across an extended SNR range (8–18 dB). The results are shown in Table 5.

As can be seen from Table 5, the positioning error does not exhibit the trend of a gradual increase with a rise in the SNR. Since only three SNR levels (−5, 0, and 5 dB) were discussed in Section 4.3 regarding the separation performance, the positioning errors at these three SNR points may not sufficiently reflect the overall trend. Additionally, we observed that the positioning errors obtained from 100 Monte Carlo simulation experiments all fall within the range of 0 m to 3.7 m, which is within the reasonable fluctuation range permitted by radar’s resolution.

(b) Noise-frequency modulation (NFM) jamming

A similar analytical approach was applied to evaluating noise-frequency modulation (NFM) jamming suppression. Figure 12, Figure 13 and Figure 14 demonstrate the anti-jamming performance of both the JADE-BSS method and the proposed method at signal-to-noise ratios (SNRs) of −5 dB, 0 dB, and 5 dB, respectively. The results show that the proposed method successfully suppresses noise and interference while maintaining target detectability even at a −5 dB SNR (Figure 12a), whereas the JADE-BSS method only achieves target detection when the SNR ≥ 0 dB (Figure 13c and Figure 14c). Moreover, the target signals separated using JADE-BSS contain significantly more residual noise and interference compared to those processed using the proposed method.

(1) The separation performance at an SNR = −5 dB

(2) Separation performance at an SNR = 0 dB

(3) Separation performance at an SNR = 5 dB

### 4.4. Evaluation of the Separation Performance Before and After Algorithm Improvements

(1) The average peak-to-sidelobe ratio (PSLR)

To evaluate the separation performance of the improved algorithm, this paper adopts the peak-to-sidelobe ratio (PSLR) of the separated target signals as the evaluation metric under two conditions: a jamming-to-signal ratio (JSR) ranging from 30 dB to 60 dB and a signal-to-noise ratio (SNR) ranging from −5 dB to 5 dB. The PSLR is defined as the amplitude ratio between the peak sidelobe and the main lobe, as expressed in Equation (25).

(25) $$PSLR=20\log_{10}\frac{SLP}{MLP}$$

where PSLR is the peak-to-sidelobe ratio of the signal. SLP is the sidelobe peak of the signal. MLP is the mainlobe peak of the signal.

To eliminate randomness in the separation results, 100 Monte Carlo simulations were conducted under both noise-amplitude modulation (NAM) suppression jamming and noise-frequency modulation (NFM) suppression jamming scenarios.

**Figure 15:**
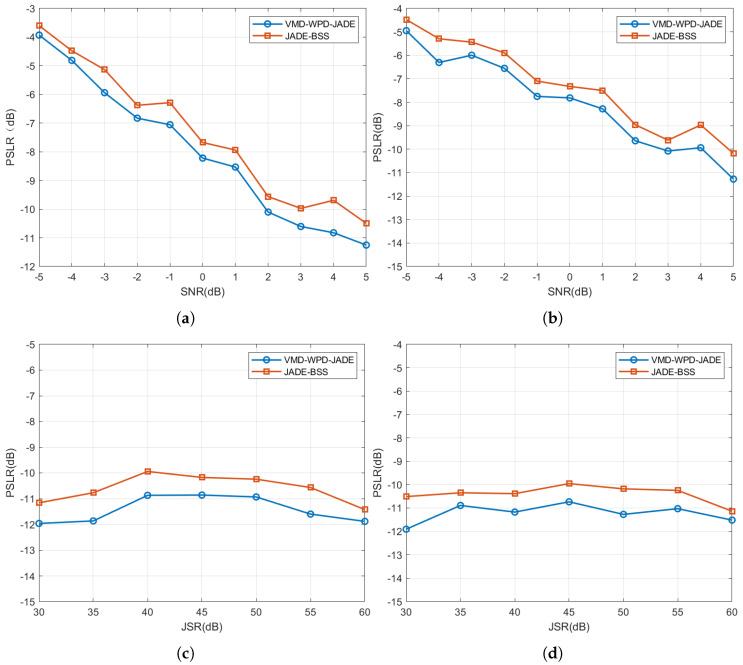
Average peak-to-sidelobe ratio (PSLR) of target signals under noise-amplitude and noise-frequency modulation suppression jamming. (**a**) Average PSLR of target signals under NAM suppression jamming (multiple SNR cases); (**b**) average PSLR of target signals under NFM suppression jamming (multiple SNR cases); (**c**) average PSLR of target signals under NAM suppression jamming (multiple JSR cases); and (**d**) average PSLR of target signals under NFM suppression jamming (multiple JSR cases).

The average peak-to-sidelobe ratios (PSLRs), calculated across an SNR range of −5 dB to 5 dB with a fixed JSR of 50 dB, are shown in Figure 15a,b. For a given JSR, the PSLRs of the target signals separated using both methods decrease gradually as the SNR increases, indicating an improved separation performance at higher SNRs. Compared to JADE-BSS, the proposed method reduces the PSLRs by 0.3284–1.1317 dB for NAM jamming and 0.4614–1.0923 dB for NFM jamming, demonstrating its superior separation capability.

Figure 15c,d present the PSLRs under a fixed SNR of 5 dB and JSRs varying from 30 dB to 60 dB. The PSLRs consistently fall within −10 dB to −12 dB, suggesting a limited impact of the jamming type and the JSR on the blind source separation performance.

(2) Detection probability (Pd)

**Figure 16:**
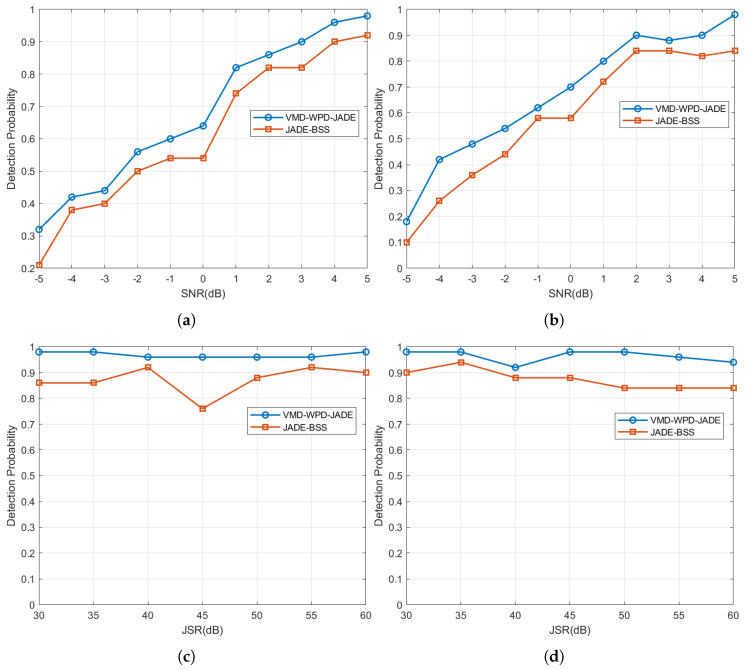
Detection probability of target signal under NAM suppression jamming and NFN suppression jamming. (**a**) Detection probability of target signal under NAM suppression jamming (multiple SNR cases); (**b**) detection probability of target signal under NFM suppression jamming (multiple SNR cases); (**c**) detection probability of target signal under NAM suppression jamming (multiple JSR cases); and (**d**) detection probability of target signal under NFM suppression jamming (multiple JSR cases).

The detection probability is defined as the ratio between the number of occurrences where the amplitude of the target signal exceeds the constant false alarm rate (CFAR) detection threshold after matched filtering and the total number of trials (set to 100 in this study). Figure 16a,b present the detection probabilities under NAM and NFM suppression jamming, respectively, with a fixed JSR of 50 dB and the SNR varying from −5 dB to 5 dB.

The simulation results demonstrate that under a jamming-to-signal ratio (JSR) of 50 dB and a signal-to-noise ratio (SNR) ranging from −5 dB to 5 dB, the target detection probability increases with a higher SNR. Compared to JADE-BSS, the proposed algorithm achieves 4–11% and 4–16% higher detection probabilities under NAM and NFM jamming, respectively, under equivalent SNR/JSR conditions. These results validate the proposed method’s superior capability to separate target signals from interference under strong jamming (a JSR = 50 dB) and low SNRs (−5 dB to 5 dB), outperforming JADE-BSS.

Additionally, Figure 16c,d compare the detection probabilities of both methods under NAM and NFM jamming with a fixed SNR of 5 dB and the JSR ranging from 30 dB to 60 dB. The proposed method maintains detection probabilities above 90%, indicating robust stability against variations in the jamming type and power within this range. In contrast, JADE-BSS exhibits lower probabilities (4–20% peak-to-peak fluctuations), confirming its inferior performance in both its detection efficacy and stability.

### 4.5. Computational Complexity Analysis

(a) An analysis of the computational complexity of each module

(1) Analysis of the computational complexity of the GWO-VMD algorithm module

The GWO-VMD (grey wolf optimizer–variational mode decomposition) algorithm integrates variational mode decomposition (VMD) and the grey wolf optimizer (GWO). In the grey wolf optimizer (GWO) parameter optimization phase, we set the population size *P* = 20 and the maximum iterations $T_{GWO}$ = 30. In the variational mode decomposition (VMD) component, the predefined parameters include the following: the decomposition mode number *K* = 15, the signal sampling points *N* = 4096, and the iteration count $T_{VMD}$ = 500. The computational complexity of variational mode decomposition (VMD) is summarized in Table 6.

Among these terms, O(KNlogN) dominates the computational load. Therefore, the overall complexity of VMD is $O_{VMD}$ = O($T_{GWO}$KNlog N) = 500×737,280≈$3.68\times 10^{8}$ operations.

The computational complexity for one GWO optimization is $O_{GWO}$ = O($T_{GWO}$P$O_{VMD}$) ≈$2.2\times 10^{11}$ operations. During the simulations, we observed that small variations in the decomposition level and the penalty factor around their optimal values had a negligible impact on the decomposition performance. Therefore, the GWO optimization process can be conducted offline through training, requiring only a single round of parameter optimization to be executed in practical applications. (2) The computational complexity of the wavelet packet decomposition (WPD) module

Wavelet packet decomposition (WPD) employs a tree-structured approach to applying multi-scale decomposition to each Intrinsic Mode Function (IMF). We set the decomposition level to *L* = 4. The computational complexity is summarized in Table 7.

Therefore, the computational complexity of wavelet packet decomposition (WPD) is $O_{WPD}$ = $K\times \left(O\left(2N2^{L}\right)+O\left(N\right)+O\left(2N2^{L}\right)\right)\approx 3.99\times 10^{6}$ operations.

(b) Overall computational complexity

The computational complexity and runtime of each module in the proposed method are presented in Table 8.

As evidenced by Table 8, the optimization time of the grey wolf optimizer (GWO) significantly exceeds that of the other algorithmic modules. This computational burden stems from two fundamental factors: Firstly, each evaluation of a candidate solution (i.e., a parameter pair [*K*,α]) requires complete execution of the variational mode decomposition (VMD) algorithm, which involves computationally intensive iterative frequency-domain decomposition and constrained optimization—a process whose complexity scales exponentially with both the signal length and the decomposition level. Secondly, the GWO’s inherent population-based mechanism necessitates maintaining substantial wolf populations and iteration counts, where every individual’s fitness evaluation in each generation compounds the overall runtime.

The runtime is dependent on the computer’s configuration, the algorithm’s complexity, and the number of signal sampling points *N*. Without compromising the anti-jamming performance, consideration may be given to appropriately reducing the number of signal sampling points and upgrading the computer hardware configurations.

## 5. Conclusions

In this study, we address the performance degradation of the conventional joint approximate diagonalization of eigenmatrices (JADE) algorithm in radar signal separation under high-jammer-to-signal-ratio (JSR) and low-signal-to-noise-ratio (SNR) conditions. To overcome this limitation, we propose a VMD-WPD-enhanced blind source separation (BSS) method for resisting mainlobe suppression jamming. The proposed method employs a VMD-WPD joint denoising algorithm to preprocess the radar echo signals, followed by whitening of the denoised signals. The whitened signals are then used to optimize the separation matrix, enabling effective extraction of the target signals via the JADE-BSS algorithm.

The simulation results demonstrate that the proposed method maintains effective separation between the target signals and noise suppression jamming even at SNR levels as low as −5 dB. Comprehensive validation through a time-domain waveform analysis, comparisons of the detection probability (showing 4–11% improvements compared to conventional JADE-BSS under NAM jamming and 4–16% under NFM jamming), and peak-to-sidelobe ratio (PSLR) evaluations (a 0.3284–1.1317 dB reduction for NAM jamming and a 0.4613–1.0923 dB reduction for NFM jamming relative to JADE-BSS) confirm the method’s robustness in low-SNR environments and its superior anti-jamming capability. Notably, the separation performance exhibits minimal sensitivity to variations in the jamming type and power level, highlighting the algorithm’s exceptional stability. This study provides an effective solution for radar systems combating mainlobe suppression jamming.

## Figures and Tables

**Table 1:** Radar parameter configuration.

Radar Parameter	Value
Bandwidth (*B*)	20 MHz
Carrier Frequency ($F_{c}$)	16 GHz
Pulse Repetition Frequency (PRF)	8 MHz
Sampling Frequency ($F_{s}$)	80 MHz
Number of Samples (*N*)	4096
Pulse Width ($T_{p}$)	25 μs

**Table 2:** Coordinate parameters of radar, target, and jammer.

Radar	Target	Jammer
(8000, 28,000, 30,000)	(10,000, 40,000, 0)	(12,000, 40,200, 1000)

**Table 3:** Optimized VMD parameters per array element.

Array Element	Decomposition Level (*K*)	Penalty Factor (α)
1	15	6492
2	15	5684
3	15	6774
4	14	6142
5	14	6251
6	14	6612

**Table 4:** Decomposition performance under different parameter combinations.

Parameter Case	α	*K*	NMSE
GWO-Optimized	6492	15	0.1211
Overestimated α	10,000	15	0.1694
Underestimated α	3000	15	0.1552
Excessive *K*	6492	20	0.1445
Insufficient *K*	6492	10	0.1694

**Table 5:** Average localization error at different SNR levels (100 Monte Carlo trials).

SNR (dB)	8	10	12	14	16	18
Error (m)	2.4094	3.3469	2.5997	2.5969	3.1594	2.9719

**Table 6:** The analysis of the computational complexity of VMD.

Computational Component	Theoretical Complexity	Actual Operations
Hilbert Transform	O (*N*log*N*)	49,152
Modal Update (Frequency Domain)	O (*KN*log*N*)	737,280
Lagrange Multiplier Adjustment	O (*N*)	4096

**Table 7:** Analysis of the computational complexity of WPD.

Computational Component	Theoretical Complexity	Actual Operations
Wavelet Packet Decomposition	O $\left(2N2^{L}\right)$	131,072
Thresholding	O (*N*)	4096
Wavelet Packet Reconstruction	O $\left(2N2^{L}\right)$	131,072

**Table 8:** Analysis of the computational complexity of the proposed method.

Module	Computational Complexity	Operations	Execution Time (s)
GWO-VMD (offline)	O ($PT_{GWO}O_{VMD})$	$2.2\times 10^{11}$	148.2127
VMD	O ($T_{VMD}KNlogN$)	$3.68\times 10^{8}$	0.68453
WPD	*K*O (65N)	$3.99\times 10^{6}$	0.24945
JADE	O ($M^{2}N$)	$1.47\times 10^{5}$	0.12725

## Data Availability

The data presented in this study are available on request from the corresponding author.
